# Targeting Trypanothione Metabolism in Trypanosomatids

**DOI:** 10.3390/molecules29102214

**Published:** 2024-05-09

**Authors:** María-Cristina González-Montero, Julia Andrés-Rodríguez, Nerea García-Fernández, Yolanda Pérez-Pertejo, Rosa M. Reguera, Rafael Balaña-Fouce, Carlos García-Estrada

**Affiliations:** 1 Departamento de Ciencias Biomédicas, Facultad de Veterinaria, Universidad de León, Campus de Vegazana s/n, 24071 León, Spain; magom@unileon.es (M.-C.G.-M.); jandrr01@estudiantes.unileon.es (J.A.-R.); ngarf@unileon.es (N.G.-F.); myperp@unileon.es (Y.P.-P.); rmregt@unileon.es (R.M.R.); 2 Instituto de Biomedicina (IBIOMED), Universidad de León, Campus de Vegazana s/n, 24071 León, Spain

**Keywords:** trypanothione, redox metabolism, oxidative stress, trypanosomatids, trypanothione reductase, trypanothione synthetase, enzyme inhibitor

## Abstract

Infectious diseases caused by trypanosomatids, including African trypanosomiasis (sleeping sickness), Chagas disease, and different forms of leishmaniasis, are Neglected Tropical Diseases affecting millions of people worldwide, mainly in vulnerable territories of tropical and subtropical areas. In general, current treatments against these diseases are old-fashioned, showing adverse effects and loss of efficacy due to misuse or overuse, thus leading to the emergence of resistance. For these reasons, searching for new antitrypanosomatid drugs has become an urgent necessity, and different metabolic pathways have been studied as potential drug targets against these parasites. Considering that trypanosomatids possess a unique redox pathway based on the trypanothione molecule absent in the mammalian host, the key enzymes involved in trypanothione metabolism, trypanothione reductase and trypanothione synthetase, have been studied in detail as druggable targets. In this review, we summarize some of the recent findings on the molecules inhibiting these two essential enzymes for *Trypanosoma* and *Leishmania* viability.

## 1. Introduction

Neglected Tropical Diseases (NTDs) represent a wide array of infectious diseases prevalent in resource-limited regions of the world, predominantly in tropical and subtropical zones [1]. Among those, Chagas disease, African trypanosomiasis (also known as sleeping sickness), and different forms of leishmaniasis are caused by trypanosomatids, a group of unicellular flagellated parasites that belong to the order Kinetoplastida [2]. These diseases are responsible for significant human suffering and economic burden [3,4], present formidable challenges to public health systems, and affect millions of people globally, particularly those residing in impoverished conditions with restricted healthcare and adequate sanitation [5]. These zoonotic diseases are transmitted by insect vectors. They are endemic to different regions of sub-Saharan African countries, Latin American countries, Asia and the Indian subcontinent, and the Mediterranean Basin, but they are becoming increasingly abundant in traditionally non-endemic areas due to the phenomena of global warming and climatic change [6].

### 1.1. Chagas Disease

Chagas disease, caused by the parasite *Trypanosoma cruzi*, is primarily transmitted to humans through the feces of triatomine bugs, commonly known as “kissing bugs” [7,8]. These insects typically feed on blood during the night and defecate near the bite wound, enabling the parasite’s entry into the body through mucous membranes or breaks in the skin [9]. Once inside the body, the parasite can induce acute symptoms, such as fever, fatigue, body aches, rash, vomiting, headache, diarrhea, and swelling at the site of infection [5,10,11]. If left untreated, it can progress to chronic stages, remaining asymptomatic in many cases. However, approximately 30% of infected individuals will develop serious complications years later, leading to heart failure [12,13] and gastrointestinal issues [14].

This disease is endemic in Latin America, affecting up to 6–7 million people, with the majority of cases concentrated in Latin America [15,16]. It predominantly afflicts rural and impoverished communities characterized by poor living conditions. Nevertheless, Chagas disease has gained global health attention due to increased migration from endemic regions to non-endemic countries [11].

### 1.2. African Trypanosomiasis

African trypanosomiasis, commonly known as sleeping sickness, is caused by two subspecies of *Trypanosoma brucei*: *T. brucei rhodesiense* and *T. brucei gambiense* [17,18]. These parasites are transmitted to humans through the bite of infected tsetse flies belonging to the genus *Glossina* [19]. The disease primarily affects populations in certain regions of central and western sub-Saharan Africa, where the tsetse fly vector is prevalent [5,17,20,21], and it can be fatal without prompt treatment [22]. African trypanosomiasis progresses through a hemolymphatic stage and a meningoencephalitis stage. In the early stage, patients may experience non-specific symptoms, including fevers, headaches, joint pains, and malaise. As the disease advances to the late stage, it affects the central nervous system, leading to neurological symptoms including disruption of the sleep cycle, altered mental status, and personality changes. If left untreated, the disease can result in coma and death [23,24,25]. 

Efforts to control African trypanosomiasis have led to a significant reduction in the number of new cases in recent years, although “blind spots” due to the lack of control and notifications over the years have been identified [26]. Despite the control efforts, African trypanosomiasis continues to pose significant public health challenges due to its high morbidity and mortality rates, particularly in rural and resource-limited settings [21].

### 1.3. Leishmaniasis

Leishmaniasis is caused by various species of the *Leishmania* parasite, including *Leishmania donovani*, *Leishmania infantum*, *Leishmania major*, *Leishmania braziliensis*, and *Leishmania mexicana*, among others. These parasites are transmitted through the bite of infected female phlebotomine sandflies, primarily belonging to the genus *Lutzomyia* in the New World and *Phlebotomus* in the Old World [27,28,29]. Leishmaniasis is widespread, occurring in parts of the tropics, subtropics, and southern Europe, with endemic regions spanning 99 countries worldwide. The epidemiology of the disease is influenced by various factors, including the climate, environmental conditions, human behavior, and the presence of suitable sandfly vectors [30,31].

Different *Leishmania* species exhibit distinct geographical distributions and clinical manifestations [32], including cutaneous, mucocutaneous, and visceral leishmaniasis, the latter being the most severe form, also known as kala-azar [33,34]. Cutaneous leishmaniasis can be caused by various species, including *L. major* in the Middle East and North Africa, *L. braziliensis* in South America, and *L. mexicana* in central and northern regions of South America [35]. It is the most common form of the disease, with approximately 700.000 to 1 million new cases reported annually [36], and it typically presents with skin ulcers [37,38]. Although less frequent, mucocutaneous leishmaniasis, which is caused by species of the *L. braziliensis* complex, namely *Leishmania panamensis* and *Leishmania guyanensis*, can have devastating effects on affected individuals, often resulting in destructive lesions affecting the mucous membranes of the nose, mouth, and throat, with severe disfigurement and disability [36]. *L. donovani* is primarily responsible for visceral leishmaniasis, particularly in the Indian subcontinent and East Africa, while *L. infantum* is associated with visceral leishmaniasis in the Mediterranean Basin, Latin America, and parts of Asia [39]. Visceral leishmaniasis is responsible for an estimated 20,000 to 40,000 deaths annually [30,40,41]. It affects various internal organs, such as the thymus, spleen, liver, and bone marrow, and it causes systemic symptoms, such as fever, weight loss, anemia, and enlarged organs [42,43,44]. A dermatological complication of visceral leishmaniasis, known as post kala-azar dermal leishmaniasis, can occur in some patients after completion of the treatment [45].

### 1.4. Current Treatments and Druggable Targets in Trypanosomatids

The number of drugs currently available to treat the diseases caused by trypanosomatids is limited, and, additionally, this therapeutic arsenal has several concerns, such as safety problems, loss of effectivity due to the emergence of resistance episodes as a consequence of overuse or misuse, and prolonged and parenteral administration protocols. The combination therapy NECT (nifurtimox and the ornithine analog α-difluoromethylornithine or eflornithine) and, recently, fexinidazole, are being used to treat sleeping sickness [46], and the nitroheterocycles nifurtimox and benznidazole have been the two drugs of choice for Chagas disease for some decades, with good results [47]. The different forms of leishmaniasis are treated with several drugs that are not exempt from problems [48]. The first-line drugs, pentavalent antimony derivatives, which have been used for almost a century, require long-time parenteral administration and present severe adverse effects in the elderly, together with the emergence of resistant strains [49]. Amphotericin B, which is a very effective drug (both deoxycholate and liposomal formulations), requires slow-infusion intravenous administration in hospitals due to its poor oral bioavailability, and it shows adverse effects, such as nephrotoxicity, hypokalemia, and myocarditis [50]. Miltefosine, which is the only oral drug with high efficacy against leishmaniasis, is embryotoxic and can easily give rise to resistant strains [51].

**Figure 1:**
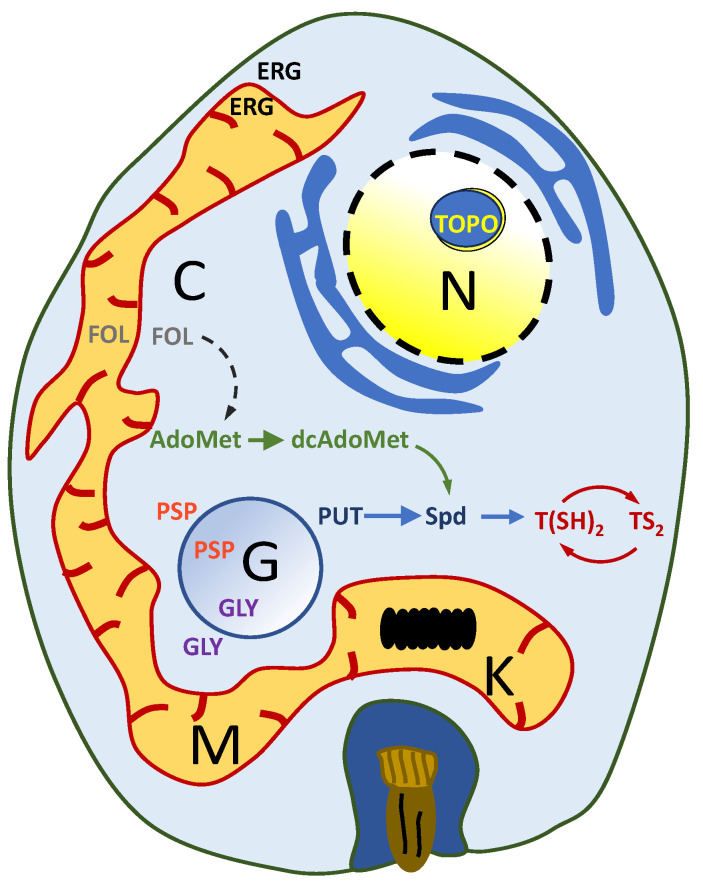
Schematic representation of a trypanosomatid amastigote. The main metabolic pathways that have been considered as potential drug targets are indicated in different colors: polyamine metabolism (blue color); methyl cycle metabolism (green color); modification of the topological state of DNA (yellow color); redox metabolism (red color); sterol biosyntyhesis (black color); folate metabolism (purple color); purine salvage pathway (orange color); folate metabolism (gray color). Abbreviations: AdoMet: S-adenosylmethionine; C: cytosol; dcAdoMet: decarboxylated AdoMet; ERG: ergosterol; FOL: folate; G: glycosome; GLY: glycolysis; K: kinetoplast; M: mitochondrion; N: nucleus; PUT: putrescine; PSP: purine salvage pathway; Spd: spermidine; TOP: DNA topoisomerase; T(SH)_2_: reduced trypanothione; TS_2_: oxidized trypanothione.

As such, there is an urgent need to find and design new promising antitrypanosomatid drugs, and different approaches, either target- or phenotypic-based, have been attempted [52]. Specific proteins and enzymes in the parasite that are essential for the viability of the parasite can be targeted to control the growth and proliferation of these parasites. Some of the druggable targets that have been more extensively studied are sterol biosynthesis, glycolysis, purine salvage pathway, DNA topoisomerases, folate metabolism, polyamine, and redox metabolism (Figure 1) [53,54,55,56]. Among these, redox metabolism and its relationship with oxidative stress are of particular interest due to the distinctive features of the redox-controlling system in trypanosomatids, which is based on trypanothione [57]. 

## 2. Oxidative Stress and the Unique Thiol Metabolism of Trypanosomatids

Oxidative stress poses a fundamental challenge for trypanosomatids, impacting their survival and pathogenicity within the host organism. These parasites, responsible for some NTDs, have evolved unique mechanisms to mitigate oxidative stress and maintain redox balance [57]. 

### 2.1. Oxidative Stress Sources

Trypanosomatids confront different environmental conditions that can induce oxidative stress [58]. This stress originates from multiple sources, including the host’s immune response, alterations in nutrient availability, fluctuations in temperature and pH, and exposure to various oxidative environments [59]. Within the host environment, trypanosomatids face a barrage of reactive oxygen species (ROS), generated by the host’s immune response, and reactive nitrogen species (RNS), as part of the oxidative burst, which is a rapid and robust production of oxidants by immune cells like macrophages and neutrophils [60]. 

In addition, the transition from the vector to a mammalian host involves significant changes in nutrient availability. Trypanosomatids must adapt to the host’s nutrient-rich environment, triggering alterations in their metabolic profile and redox balance [61]. Swift changes in glucose, amino acids, and lipid availability can impact mitochondrial respiration, glycolysis, and antioxidant defenses, influencing the production and detoxification of ROS/RNS [62]. Furthermore, certain host tissues create oxidative microenvironments that challenge the antioxidant capacity of trypanosomatids. These parasites must contend with these hostile oxidative conditions while establishing infection and evading immune detection. Moreover, the temperature and pH fluctuations between the vector and the host can modulate the activity of antioxidant enzymes and the production of ROS/RNS [63]. These fluctuations serve as cytotoxic molecules that target trypanosomatids, causing oxidative damage to their cellular components, including lipids, proteins, and DNA, leading to cell death [64] and disrupting essential metabolic pathways. As such, effective management of oxidative stress becomes indispensable for the parasite’s survival within the host [8,57,65].

### 2.2. Defense against Antioxidants in Trypanosomatids: Trypanothione as a Key Player

**Figure 2:**
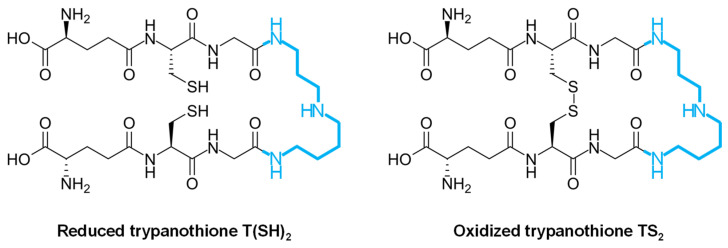
Biochemical structure of trypanothione in the reduced [T(SH)_2_] and oxidized [TS_2_] forms. The spermidine molecule joining two molecules of glutathione is highlighted in blue.

Unlike the mammal host, infective trypanosomatids lack catalase, Se-dependent glutathione peroxidase, glutathione reductase, and thioredoxin reductase [7,66,67,68]. Instead, trypanosomatids possess a unique antioxidant defense mechanism centered around the peptide molecule trypanothione [69,70,71], thereby relying on the trypanothione reductase (TryR) system rather than the glutathione–glutathione reductase system to manage oxidative stress [7]. Trypanothione is a low-molecular-weight dithiol (N1,N8-bis(glutathionyl)spermidine) that contains two molecules of glutathione joined by a spermidine linker [7,67,69,71] (Figure 2), and it serves as a pivotal player in maintaining redox balance and combating oxidative damage within these parasites. 

**Figure 3:**
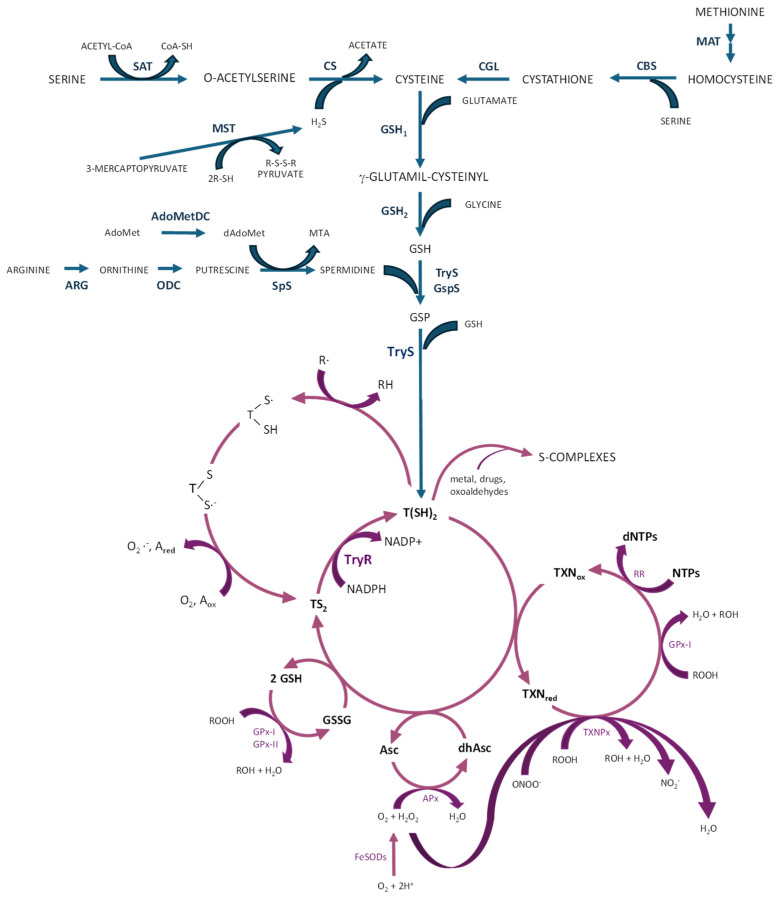
Biosynthesis of trypanothione (blue color) and antioxidant pathways (purple color) in trypanosomatids. AdoMetDC: S-adenosylmethionine decarboxylase; APx: ascorbate-dependent heme-peroxidase; ARG: arginase (not in *T. cruzi* and *T. brucei*); CBS: cystathione beta-synthase; CGL: cystathionine gamma-lyase; CS: cysteine synthase; FeSODs: iron–superoxyde dismutases; GPx-I and GPx-II: non-selenium glutathione peroxidases; GSH: reduced glutathione; GSH1: γ-glutamylcysteine synthetase; GSH2: glutathione synthetase; GSP: mono-glutathionylspermidine; GspS: GSP synthetase; GSSG: oxidized glutathione; MAT: methionine adenosyl transferase; MST: mercaptopyruvate sulfurtransferase; ODC: ornithine decarboxylase (not in *T. cruzi*); RR: ribonucleotide reductase; SAT: serine acetyltransferase; SpS: spermidine synthase; TryR: trypanothione reductase; TryS: trypanothione synthetase; TXNPx: tryparedoxin peroxidase (figure adapted from [7,8,71]).

In trypanosomatids, most of their glutathione is converted into trypanothione, which in turn may spontaneously reduce tryparedoxin (a trypanosomatid-specific thioredoxin-like protein containing two cysteines at the amino terminal end) and dehydroascorbate. Tryparedoxin peroxidase reduces various types of peroxides using electrons donated by tryparedoxin, which is restored to the reduced state by trypanothione [66]. The trypanothione system orchestrates a sophisticated defense mechanism against ROS/RNS; therefore, it acts as the key reductant within these parasites, serving as a reducing agent for various enzymes engaged in DNA synthesis, detoxification of hydroperoxides, and reduction of methionine sulfoxides, thus protecting the parasites from oxidative burst occurring during host infection, which is crucial for the survival and virulence of these parasites [7,70,72]. Trypanothione directly reduces tryparedoxin, dehydroascorbate, and glutathione disulphide through sequential reactions coupled with the reductive detoxification of peroxides and the formation of deoxyribonucleotides. The oxidized trypanothione (disulphide form or TS_2_) is transformed back into the reduced form of trypanothione T(SH)_2_ by trypanothione reductase (TryR) in the presence of NADPH as an electron donor [65,67,73,74]. In addition to TryR, several enzymes contribute to the defensive mechanisms in trypanosomatids against oxidative stress (Figure 3). They are located on different cellular compartments and become activated in response to oxidizing agents [65]. 

Iron–superoxide dismutases (FeSODs) neutralize superoxide radicals (O_2_^−^) produced within cytosol (FeSOD-B1), mitochondria (FeSOD-A and C), and glycosomes (FeSOD-B1-2), transforming them into H_2_O_2_ and O_2_ [61,71]. In trypanosomatids, several peroxidases have been identified, each with differing subcellular localization and substrate specificities [75]. GPX-I, a non-selenium glutathione peroxidase, is situated in both the cytosol and glycosome, whereas GPX-II is present in the endoplasmic reticulum. They confer resistance against hydroperoxides and lipid hydroperoxides, respectively, and use glutathione and/or tryparedoxin as substrates for reduction [8]. Cytosolic and mitochondrial tryparedoxin peroxidase (c- or m-TXNPx) from the 2-cysteine peroxiredoxin family can detoxify peroxynitrite (ONOO^−^), H_2_O_2_, and small-chain organic hydroperoxides (ROOH) using tryparedoxin [7,68], facilitating the conversion of ROOH into their corresponding alcohols (ROH), ONOO^−^ into NO_2_^−^ and H_2_O_2_ into H_2_O. Tryparedoxin further transfers electrons to ribonucleotide reductase, facilitating the production of the deoxyribonucleotides crucial for DNA synthesis [7,8,55,61,71] (Figure 3).

### 2.3. Trypanothione Biosynthesis and the Trypanothione Redox System

The precursor molecules for the biosynthesis of trypanothione are glutathione and spermidine [7,76]. Glutathione is formed by the combination of glutamate, glycine, and cysteine in a reaction catalyzed by γ-glutamylcysteine synthetase and glutathione synthetase [61,65,77]. The polyamine spermidine has to be synthesized from its amino acid precursors, L-arginine and L-methionine [56,78], or otherwise internalized from the host by specific transporters [79]. In *T. brucei* and *Leishmania*, spermidine is derived from ornithine, which is formed from L-arginine due to the activity of arginase in *Leishmania*, but not in *T. brucei* and *T. cruzi*, which lack this enzymatic activity [80]. Ornithine is transformed into putrescine by ornithine decarboxylase, and after conjugation of putrescine with an aminopropyl group from decarboxylated S-adenosyl-L-methionine (a metabolic intermediate derived from the condensation of L-methionine with ATP) [81], spermidine is formed in a reaction catalyzed by spermidine synthase (Figure 3). *T. cruzi* lacks the gene-encoding ornithine decarboxylase and, as such, putrescine has to be taken up by the parasite [78].

The biosynthesis of trypanothione is achieved after the ATP-dependent addition of two glutathione molecules to the extreme amino groups of spermidine. This reaction can be catalyzed, depending on the trypanosomatid species, by trypanothione synthetase (TryS, EC 6.3.1.9) or the subsequent action of mono-glutathionylspermidine synthetase (GspS, EC 6.3.1.8) and TryS, with the production of trypanothione, ADP, and phosphate. While GspS is specialized in the formation of mono-glutathionylspermidine from glutathione and spermidine, TryS can accept, in addition to glutathione, both spermidine and mono-glutathionylspermidine as substrates to synthesize either mono-glutathionylspermidine (reaction intermediate) or T(SH)_2_, respectively [71,82]. GspS activity has not been detected in *L. infantum*, and knock-out parasites in the gene encoding this protein did not show impaired viability. On the contrary, TryS is essential in this parasite [83], *T. brucei* [84], and *T. cruzi* [85]. TryS is a cytosolic monomeric protein with a molecular weight of 69–79 kDa, and it has two catalytic domains. The synthetase C-terminal domain has a feature structure usually found in carbon–nitrogen ligases, an ATP-grasp family fold, with the active site forming a cavity with a triangular shape accommodating each of the three substrates (ATP, spermidine or glutathionylspermidine, and glutathione) [86]. This domain uses the energy coming from ATP hydrolysis to promote the conjugation of glutathione and spermidine, enabling the formation of the intermediate form, glutathionylspermidine, and, finally, T(SH)_2_, the end product [71,76,77,87]. The N-terminal cysteine, the histidine-dependent aminohydrolase amidase domain, is involved in the conversion of gluthathionylspermidine and trypanothione back to the original substrates [71,76]. Therefore, this crucial enzyme can catalyze the reverse reaction (cyclic renewal or replacement of trypanothione), although at a much lower rate [71]. 

After synthesis, T(SH)_2_ runs within the cytosol to actively reduce disulfides in proteins (RS2) like thioredoxin, tryparedoxin, glutathione disulfide, and dehydroascorbate [65]. Upon reduction of dehydroascorbate, ascorbate is used as substrate for ascorbate-dependent heme-peroxidase (APx), located on the endoplasmic reticulum, which provides resistance against H_2_O_2_ challenge [7,8,65]. T(SH)_2_ has the ability to react with radical species (R-) in repair or scavenging reactions, producing trypanothione thiol radicals [7,8,71]. The sulfur-centered radical combines with the neighboring thiol to generate a trypanothione disulfide anion radical, which transforms into the stable TS_2_ through the generation of secondary radicals (e.g., O_2_^−^). Furthermore, T(SH)_2_ is able to bind with metal-containing drugs (S-complexes), which could be sequestered within the cell or extruded, likely via specialized transporters (Figure 3) [65,72].

As mentioned above, as a consequence of the reduction reactions, the resulting TS_2_ has to be reduced back to its active dithiol state through the action of NADPH-dependent flavoenzyme TryR. This enzyme has an essential role in the survival and proliferation of these parasites within the host environment, which has been demonstrated both in vitro and in the context of human infections. In fact, TryR-deficient trypanosomes (knock-out mutants) exhibit avirulence and heightened susceptibility to oxidative stress [67,73,82]. 

The crystal structure of TryR from several trypanosomatids (108–112 kDa) reveals that this enzyme is a homodimer, with each of the two subunits comprising a FAD-binding domain, a NADPH-binding domain, and an interface domain, which is responsible for the homodimer assembly [74,88,89,90]. The enzyme contains two cavities, one for each substrate (NADPH and TS_2_), which face opposite sides of the isoalloxazine ring of FAD. The TS_2_ site is located at the interface between the two subunits, and the reaction occurs through the transfer of two electrons from NADPH, via the FAD cofactor, to two catalytic cysteines (Cys52 and Cys57). TS_2_ binds the protein after reduction of these two residues, and because of the attack of Cys52 (deprotonated by the couple His461’-Glu466’) on the disulfide bridge of the substrate and Cys57 on Cys52, the T(SH)_2_ is released [91]. Unlike the glutathione-binding domain of glutathione reductase, the TS_2_-binding site of TryR contains a wider hydrophobic negative charge region; as such, this enzyme represents a selective target [92].

TryR exhibits a dual cytosolic and glycosomal distribution within the cell. The cytosolic fraction enables TryR to engage with various cellular components, whereas the glycosomal constituent provides a unique milieu for redox reactions. Notably, in *T. brucei*, TryR has been found exclusively in the cytosolic fractions. This localization pattern suggests distinct regulatory mechanisms and functional adaptations in these parasites [7].

## 3. Targeting Trypanothione Reductase and Trypanothione Synthetase in Trypanosomatids

The multifaceted nature of trypanothione metabolism extends beyond its role in antioxidant defense. It is intricately linked to other essential cellular processes, such as DNA synthesis, detoxification of hydroperoxides, and reduction of methionine sulfoxides, highlighting its significance in the overall physiology of trypanosomatids. Therefore, drugs that can inhibit this system could potentially increase the susceptibility of the parasites to the host’s defense mechanisms and favor their elimination [55,73,74,93,94]. Due to the key role of TryR and TryS in the metabolism of trypanothione in trypanosomatids, different inhibitors have been tested against these enzymes as potential antitrypanosomatid compounds.

### 3.1. Inhibitors of Trypanothione Reductase

The absence of the TryR system in mammals emphasizes the evolutionary divergence between these organisms and their adaptation to distinct ecological niches. The unique reliance of trypanosomatids in the TryR system presents an enticing opportunity for therapeutic intervention [57]. Because this system is vital for the parasite’s survival but absent in mammalian hosts, it emerges as a promising target for the development of novel drugs against trypanosomatid diseases. Several classes of active compounds that inhibit this enzyme have been described, and this topic has been the subject of extensive reviews [91,95]. The majority of inhibitors characterized so far bind to the wide TS_2_ cavity, whereas other compounds have been reported to bind to the NADPH cavity or to Cys52 and Cys57 in the catalytic site [91]. Disassembly of the dimer has been described as another mechanism of inhibition, and some compounds have been described to act as mixed inhibitors or “subversive” substrates. Summarized information is indicated below.

#### 3.1.1. Inhibitors Binding to the Wide TS_2_ Cavity (Competition with Trypanothione) 

**Figure 4:**
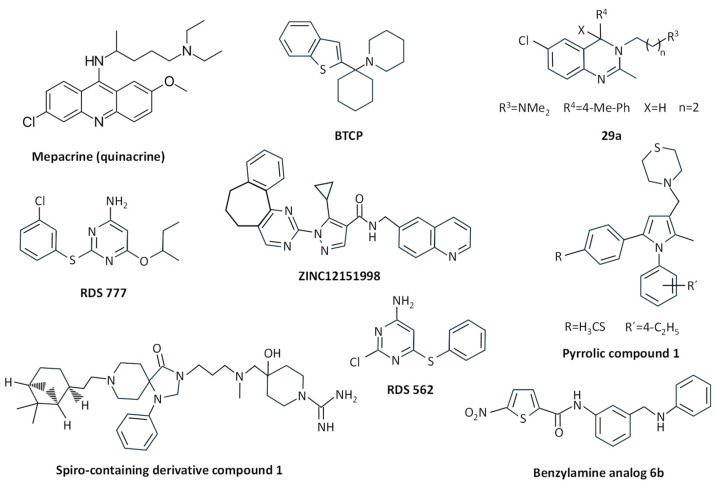
Chemical structure of different TryR inhibitors binding to the wide TS_2_ cavity.

Early studies described the structure of the complex between *T. cruzi* TryR (EC 1.6.4.8) and the antiparasitic drug mepacrine (quinacrine) (Figure 4) [96], and several derivatives and structural analogues of this compound have been characterized in terms of kinetics and their structure–activity relationship [97,98,99]. Sulfonamide and urea analogues of quinacrine showed better TryR inhibition properties than the parental molecule, and urea derivatives exhibited good in vitro activity against *T. brucei*, *T. cruzi*, and *L. donovani*, among other parasites [100]. The combination of the structural motif of mepacrine with diaryl sulfides improved the binding efficacy of the resulting conjugates, providing good in vitro activity against *T. brucei rhodesiense* and *T. cruzi* [101].

The arylcyclohexylamine BTCP (1, 1-(1-benzo[b]thiophen-2-yl-cyclohexyl)-piperidine) (Figure 4), identified after high-throughput screening of compounds against *T. cruzi* TryR [102], was reported to inhibit TryR from *T. brucei* in a competitive way, showing activity against bloodstream forms of *T. brucei*, and it is considered an interesting compound for further development despite its poor selectivity against mammalian cells [103]. In fact, several improved derivatives were obtained through a combination of structural motifs of imidazole-based diaryl sulfides and BTCP-based TryR inhibitors, thus giving rise to more potent and high-selectivity analogues with activity against *T. brucei rhodesiense* in vitro and low cytotoxicity to mammalian cells [104]. Further improvement steps have been performed over these analogues through the introduction or modification of substituents [105], such as the improvement of the indole N-substituent and the introduction of a propargylic substituent in position 4 of the thiazole moiety, which provided increased binding affinity towards *T. brucei* TryR and trypanocidal activity [106].

Compounds with a 3,4-dihydroquinazoline core structure, which were derived from hit compounds previously identified from a high-throughput screening against *T. cruzi* [107], were able to show improved inhibition of both TryR and *T. cruzi* growth, the most potent inhibitor being the C4 substituted compound 3-(6-Chloro-2-methyl-4-p-tolylquinazolin-3(4*H*)-yl)-*N*,*N*-dimethylpropan-1-amine (29a) (Figure 4). Despite this improvement, the authors suggested further optimization to improve cellular potency and selectivity [108]. 

The pyrrolic compound 1 (4-((1-(4-ethylphenyl)-2-methyl-5-(4-(methylthio)phenyl)-1H-pyrrol-3-yl)methyl)thiomorpholine) (Figure 4), chosen from an in-house library of compounds that inhibited *L. donovani* growth, was reported to competitively inhibit *L. infantum* TryR, having a bioactivity similar to pentavalent antimony against *L. donovani* amastigotes [89].

After the molecular docking of twenty molecules from a ZINC database, compound ZINC12151998 (an *N*-(6-quinolinemethyl)-3-pyrazole carboxamide) (Figure 4) was found to inhibit 32.9% of the activity of the recombinant *L. major* TryR. This molecule showed good antileishmanial activity, and molecular dynamics studies showed interaction of this compound with the enzyme active site [109].

From a collection of 3097 antitrypanosomatid compounds, the spiro-containing derivative compound 1 (4-(((3-(8-(2-((1S,2S,5S)-6,6-dimethylbicyclo [3.1.1]heptan-2-yl)ethyl)-4-oxo-1-phenyl-1,3,8 triazaspiro [4.5]decan-3-yl)propyl)(methyl) amino)methyl)-4-hydroxypiperidine-1-carboximidamide) (Figure 4) was selected due to its in vitro potency and high solubility. This compound was competitive with TS_2_ for both *T. brucei* and *L. infantum* recombinant TryR, inhibited the endogenous *T. brucei* enzyme while being inactive on human glutathione reductase, and showed bioactivity against *T. brucei* in the low micromolar range [110].

Within an in-house library of diaryl sulfide thioether derivatives previously synthesized as anti-HIV agents, compound RDS 777 (6-(sec-butoxy)-2-((3-chlorophenyl)thio) pyrimidin-4-amine) (Figure 4) was able to exert antiproliferative effects on *L. infantum* promastigotes in the micromolar range. This was due to the inhibition of TryR with high efficiency by binding with high affinity to the catalytic site of this enzyme, thus inhibiting TS_2_ binding and its reduction, and also to the NADPH-binding site [111]. The structurally related diaryl sulfide analog RDS 562 (Figure 4) showed better leishmanicidal properties, and although it did not inhibit the human glutathione reductase, it was less effective in inhibiting TryR than RDS 777 [112].

Synthesis of selenocyanate and diselenide derivatives containing amide moieties gave rise to the selection of three molecules with high potency against *L. infantum* either in culture or as intramacrophagic amastigotes, showing high selectivity in human THP-1 monocytic cells: *N,N′*-(4,4=-diselanediylbis [4,1-phenylene])bisfuran-2-carboxamide (2h), *N,N′*-(4,4=-diselanediylbis [4,1-phenylene])bisadamantamide (2k), and *N,N′*-(4,4=-diselanediylbis [4,1-phenylene])bisnaphthamide (2m). These compounds exhibited good inhibitory activities against TryR, and although no structural studies were provided to define their mode of action, it was suggested to occur by binding the enzyme and competing with the substrate [113].

Inhibition of TryR has been suggested to be one of the mechanisms of action of some nitroheterocyclic compounds. The 7-nitroquinoxalin-2-one derivative VAM2-4 (7-nitro-4-[5-(1,2,3,4-tetrahydroisoquinolin-2yl)pentyl]-3,4-dihydro-1H-quinoxalin-2-one hydrobromide) was active against *T. cruzi* and inhibited TryR through the interaction with His461 and Glu466, the latter being involved in the enzyme catalytic activity [114]. Several 5-Nitrothiophene-2-carboxamide derivatives were synthesized from *N*-{4-methoxy-3-[(4-methoxyphenyl)sulfamoyl]phenyl}-5-nitrothiophene-2-carboxamide, the latter being identified as the best antileishmanial compound within a set of 192 molecules selected by GlaxoSmithKline starting from the LeishBox. The most effective derivative inhibiting TryR was the benzylamine analog 6b (Figure 4), which was reported to bind to the TS_2_-binding site through a competitive mechanism, and it caused *L. infantum* promastigote and amastigote death at micromolar concentrations with a high selectivity index [115].

By means of a fragment-based drug discovery strategy, new TryR inhibitors have been developed. From these, compounds 9 (4-(((5-((4-Fluorophenethyl) carbamoyl) furan-2-yl) methyl) (4-fluorophenyl) carbamoyl)-1-methyl-1-(3-phenylpropyl) piperazin-1-iumIodide), 10 (1-(3,4-Dichlorobenzyl)-4-(((5-((4-fluorophenethyl) carbamoyl) furan-2-yl) methyl) (4-fluorophenyl) carbamoyl)-1(3-phenylpropyl) piperazin-1-iumIodide), and 14 (1-(3,4-Dichlorobenzyl)-4-(((5-((4-fluorophenethyl) carbamoyl) furan-2-yl) methyl) carbamoyl)-1-(3-phenylpropyl)p iperazin-1-iumIodide) were the best inhibitors of TryR by anchoring to the so-called Z-site, thus competing with TS_2_ and potentially impeding its entrance into the cavity. Compound 10 was the best inhibitor, whereas compound 9 showed the best antileishmanial effects in vitro and ex vivo [116].

#### 3.1.2. Metal Binding to Cys52 and Cys57 in the Catalytic Site 

**Figure 5:**
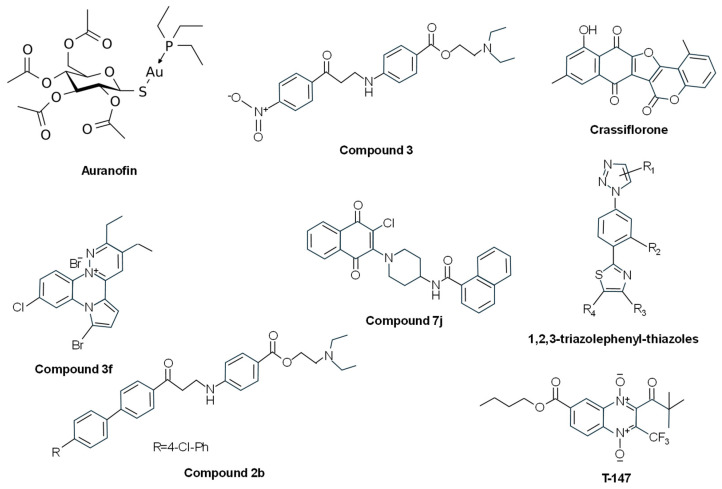
Chemical structures of different TryR inhibitors binding to the Cys52 and Cys57 in the catalytic site, or the NADPH-binding cavity, or disassembling the dimer, or acting as mixed inhibitors.

Several metal complexes have been tested on TryR. Early studies reported that antimony can inhibit this enzyme in intact cells [117] through the formation of a complex between Sb(III) and the residues of the catalytic triad (Cys52, Cys57, and His461) [118]. Silver can also inhibit TryR at nanomolar concentrations. This metal is more effective than antimony in vitro, showing a mechanism of inhibition very similar to that of Sb(III) and inducing antiproliferative effects at micromolar concentrations on *L. infantum* treated with nanoparticles encapsulated by ferritin molecules (PfFt-AgNPs) [119]. Similar inhibitory results on TryR have been obtained with gold-containing compounds [120], such as gold(1+); (2*S*,3*R*,4*S*,5*R*,6*R*)-3,4,5-triacetyloxy-6-(acetyloxymethyl)oxane-2-thiolate;tri-ethylphosphane (auranofin) (Figure 5). This gold salt classified by the WHO as an antirheumatic agent showed antileishmanial effects at micromolar concentrations and seemed to inhibit the enzyme through a dual mechanism where the gold atom binds to Cys52 and Cys57 of the active site and the thiosugar moiety binds to the trypanothione-binding site [121]. Other antitrypanosomatid compounds based on palladium and platinum have been reported to irreversibly inhibit TryR [122,123].

#### 3.1.3. Inhibitors Binding to the NADPH-Binding Cavity (Competition with NADPH) 

Some TryR inhibitors have been reported to bind the NADPH-binding cavity. In addition to the diaryl sulfide thioether derivative RDS 777 indicated above [111], other antileishmanial compounds share this mechanism of action. Through a new luminescent-based HTS assay performed with an in-house library of 120,000 compounds, 2-(diethylamino)ethyl-4-((3-(4-nitrophenyl)-3-oxopropyl)amino)benzoate (compound 3) (Figure 5) was identified. This molecule was able to inhibit TryR by binding the enzyme in a cavity at the entrance of the NADPH-binding site. It did not inhibit the human glutathione reductase, and it presented dose-dependent cytotoxic effects against *L. infantum* promastigotes in the low micromolar range, thus representing a good starting point for the design of new antileishmanial drugs [124]. Based on these results, new 3-amino-1-arylpropan-1-one derivatives structurally related to compound 3 were synthesized. Among them, 2-(diethylamino)ethyl4-((3-(4’-chloro-[1,1’-biphenyl]-4-yl)-3-oxopropyl)amino)benzoate (compound 2b) (Figure 5), which bears a 4-chlorophenyl group in position 4 of the acetophenone moiety, was found to keep the inhibitory activity against *L. infantum* TryR in the micromolar range by binding to the NADPH cavity entrance, thus interacting with Tyr221, Arg228, and Gly195 and impeding the NADPH entrance [94]. 

#### 3.1.4. Inhibitors Disassembling the Dimeric Structure of TryR

After the analysis of the dimerization interface of *L. infantum* TryR, residue E436 was validated as a hot spot for structural integrity of the dimer and catalytic activity. Short linear and cyclic peptides (P3: Ac-PEIIQSVGISNLKNL-NH2 and P4: Ac-PKIIQSVGISNLKNL-NH2) derived from an α-helix structure spanning residues P435 to M447 were able to strongly affect enzyme dimerization and activity [125]. Later, it was observed that P4 acts as a pseudoirreversible, time-dependent, non-competitive inhibitor of the enzyme [126].

In another study using a proteomimetic approach, two types of nonpeptidic small-molecule dimerization disruptors were synthesized in order to improve drug-like properties. The pyrrolopyirimidine 4-(*N*-(2-Amino-2-oxoethyl)-7-(3-amino-3-oxopropyl)-4-(dimethylamino)-2-((2-(naphthalen-2-yl)ethyl)amino)-7Hpyrrolo [2,3-d]pyrimidine-6-carboxamido)butan-1-aminium 2,2,2-trifluoroacetate (compound 2f) was described as a novel class of bicyclic and heterocyclic inhibitor as it was inactive on dimerization and binds the mepacrine-binding site, showing moderate inhibition of the oxidoreductase activity. The other type of small molecules based on the imidazole-phenyl-thiazole scaffold, represented by N1-((1-(4-(4-(2-(Naphthalen-2-yl)ethyl)thiazol-2-yl)-3-(2-(2-oxoimidazolidin-1-yl)ethoxy)phenyl)-1H-imidazol-2-yl)-methyl)ethane-1,2-diaminium 2,2,2-Trifluoroacetate (compound 3e) and N1-((1-(4-(4-(2-([1,1′-Biphenyl]-4-yl)ethyl)thiazol-2-yl)-3-(2-(2-oxoimidazolidin-1-yl)ethoxy)phenyl)-1H-imidazol-2-yl)methyl)ethane-1,2-diaminium 2,2,2-Trifluoroacetate (compound 3f), behaved as dissociative inhibitors and were better than the pyrrolopyrimidine-based derivatives in terms of enzyme inhibition and antileishmanial properties [127]. Later, these authors evaluated novel 1,2,3-triazolephenyl-thiazole compounds (Figure 5), and those bearing (poly)aromatic substituents were found to behave as slow-binding, non-competitive inhibitors endowed with high antileishmanial activity [128]. 

#### 3.1.5. Mixed Inhibitors

Several compounds with different chemical structures have been described to act as mixed inhibitors, i.e., they bind the enzyme whether or not the enzyme has already bound the substrate. The pentacyclic furocoumarin naphthoquinone crassiflorone (Figure 5) was used as a parental molecule for the development of novel dual glyceraldehyde-3-phosphate dehydrogenase/TryR inhibitors. One of these derivatives, (11-Hydroxy-6H-naphtho [2’,3’:4,5]furo [3,2-c]chromene-6,7,12-trione) (compound 19), was able to inhibit both enzymes with a balanced profile (64% *T. brucei* glyceraldehyde-3-phosphate dehydrogenase and 65% *T. cruzi* TryR). This compound exhibited a mixed-type inhibition on the recombinant *T. cruzi* TryR but a negligible inhibition of the growth of *L. infantum*, *T. brucei*, and *T. cruzi* [129]. After the synthesis of several n-butyl and isobutyl quinoxaline-7-carboxylate 1,4-di-N-oxide derivatives, one of these compounds (T-147) (Figure 5) showed good trypanocidal activity against *T. cruzi* and behaved as a mixed-type inhibitor of *T. cruzi* TryR, although it also behaved as a non-competitive inhibitor of human glutathione reductase [130]. The naphthoquinone derivative *N*-(1-(3-chloro-1,4-dioxo-1,4-dihydronaphthalen-2-yl)piperidin-4-yl)-1-naphthamide (Compound 7j) (Figure 5) showed good trypanocidal activity on epimastigotes and trypomastigotes of *T. cruzi* with a high selective index. This compound behaved as a simple mixed-type inhibitor towards TS_2_ and NADPH, thus suggesting that 7j and substrates bind to different sites of *T. cruzi* TryR [131]. 

From fifteen pyridazino-pyrrolo-quinoxalinium salts, the derivative 7-bromo-10-chloro-2,3-diethyl-8H-pyridazino [2,3-a]pyrrolo [2,1-c]quinoxal-13-inium bromide (compound 3f) (Figure 5) was selected due to its potent antileishmanial activity. This compound showed hyperbolic mixed inhibition of *L. infantum* TryR in the presence of saturating concentrations of NADPH, hyperbolic non-competitive inhibition of *L. infantum* TryR versus TS_2_, and hyperbolic uncompetitive behavior at varying concentrations of NADPH, acting as a “subversive” substrate (see below) for this enzyme [132].

Recently, two compounds, ZINC1033681 (Zn_C687) and ZINC10213096 (Zn_C216), selected after virtual screening of 2163 phenothiazine derivatives, were able to decrease *T. cruzi* growth by 20% and 50%, respectively, and inhibited the recombinant TryR in a mixed-type manner [133]. 

#### 3.1.6. “Subversive” Substrates (Turncoat Inhibitors)

Uncompetitive TryR inhibitors may behave as “subversive” substrates, not only altering the physiological reduction of TS_2_ but also converting TryR in a prooxidant enzyme that produces O_2_^−^ through a futile consumption of NADPH and O_2_. Early studies showed that TryR can catalyze one-electron reduction of naphthoquinone and nitrofuran derivatives [134]. 

**Figure 6:**
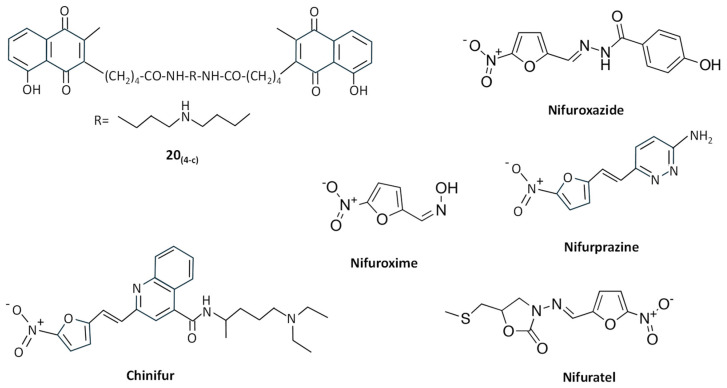
Chemical structures of different “subversive” substrates for TryR.

Menadione and other related 1,4-naphthoquinones act as redox cyclers or “subversive” substrates for TryR [135]. Different 2- and 3-substituted 1,4-naphthoquinone derivatives were synthesized, and compound 20_(4-c)_ (Figure 6) proved to be a potent “subversive” substrate for *T. cruzi* TryR and an effective uncompetitive inhibitor versus TS_2_ and NADPH, showing potent activity against *T. brucei* and *T. cruzi* [136].

Nitrofuran derivatives cause irreversible inactivation of TryR under anaerobic conditions, whereas in the presence of O_2_, instead of inactivating the enzyme, they behave as “subversive” substrates causing the production of free radicals, inhibiting the reduction of TS_2_, and leading to futile consumption of NADPH, which may provoke detrimental effects in *T. cruzi* [134]. For example, 2-{5′-nitro(furo-2′-yl)-ethene-1-yl}-4(N,N-diethylamino)-1-methyl-but-1-yl-arninocarbonyl-4-quinoline (chinifur) (Figure 6) has been reported to be the most selective inhibitor of, and free-radical-generating substrate for, TryR from *T. congolense* [137], having a potent inhibition on TS_2_ reduction in *T. cruzi* [138]. Like other nitrofurans with aromatic and heterocyclic substituents, chinifur acted as an uncompetitive inhibitor for NADPH and as a non-competitive or uncompetitive inhibitor for TS_2_ [137]. Nifuroxazide, nifuroxime, and nifurprazine (Figure 6) were the most effective “subversive” substrates of *T. cruzi* TryR, with nifuroxazide and nifurprazine showing antiparasitic activity in the low micromolar range [138]. Other nitrofuran derivatives, including (5-Nitro-furan-2-carboxylic acid (3-dimethylamino-propyl)-amide, 5-Nitro-furan-2-carboxylic acid benzylamide, and 5-Nitro-furan-2-carboxylic acid dibenzylamide), followed a linear, reversible, uncompetitive inhibition pattern of TryR and showed toxicity against *T. cruzi* epimastigotes at low micromolar concentrations [139]. More recently, the nitrofuran derivative nifuratel (Figure 6) was identified as a strong antileishmanial compound from a repurposing library of anti-infectious drugs [140]. This compound, in combination with miltefosine, has shown synergic antileishmanial effects both in vitro and in vivo, inhibiting TryR in an uncompetitive way [47,141].

### 3.2. Inhibitors of Trypanothione Synthetase

Although less effort has been directed to developing TryS inhibitors, TryS has been validated as a drug target in different trypanosomatids, and information provided by TryS screening programs has been considered useful for the identification of new TryS inhibitors by means of the computer-aided drug discovery, which is a key strategy for the quick and cost-efficient identification of new active scaffolds [142]. Several compounds have been reported to inhibit TryS, from substrates or transition state analogs to natural and synthetic compounds, as reviewed elsewhere [92,95,143]. Summarized information is indicated below.

#### 3.2.1. Substrate or Transition State Analogs

Initial studies carried out before the crystal structure of TryS was known were conducted with *N*-benzoyloxycarbonyl-*S*-(2,4-dinitrophenyl) glutathione derivatives and phosphinic acid pseudopeptides (mimics of a presumed transition state in trypanothione biosynthesis). Despite their good potency in *Crithidia fasciculata*—a model of trypanosomatid with mosquitoes as definitive hosts—these compounds did not work well against TryS from pathogenic trypanosomatids and showed disappointing results in cells due to their peptide nature [144]. Similar results were obtained with paullone 7,12-dihydrobenzo [2,3]azepino [4,5-b]indol-6(5*H*)-one derivatives, a class of ATP analogs targeting the ATP-binding cassette in protein kinases. A single non-catalytic residue exchange modifying the interaction of these compounds with the enzyme is responsible for this difference in sensitivity between *C. fasciculata* and pathogenic trypanosomatids TryS [82,144]. 

**Figure 7:**
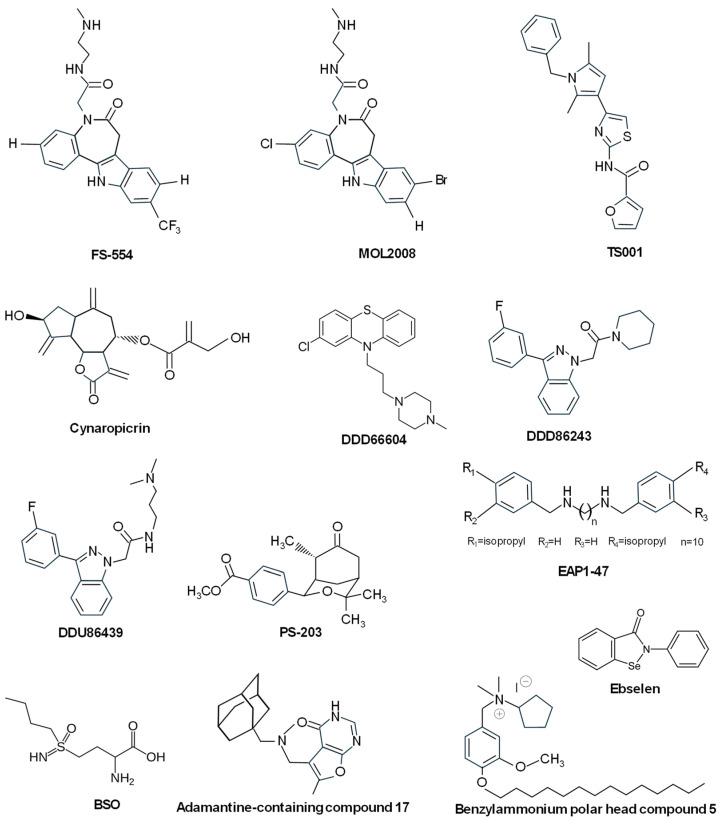
Chemical structures of different TryS inhibitors.

Related to this chemical structure, 9- and 11-substituted 4-azapaullones were efficient in killing *T. brucei* parasites, although they exhibited a modest inhibition of the *T. brucei* TryS [145]. On the contrary, the N^5^-substituted paullone FS-554 (Figure 7) showed good inhibitory properties of *L. infantum* TryS, together with antiparasitic activity against promastigotes, this activity being inversely correlated with the expression levels of TryS, thus supporting the on-target effect of the paullone [83]. Another N^5^-substituted paullone (MOL2008) (Figure 7) proved to be almost one order of magnitude more potent towards *L. infantum* promastigotes than FS554, inhibiting TryS and targeting in vivo trypanothione biosynthesis [146]. These results revealed that substitution of 4-azapaullones in position 9 or 11 with alkyl or aryl groups is detrimental for target inhibition, whereas the addition of a *N*-[2-(methylamino)ethyl]acetamide side chain at the N^5^-position of 9-trifluoromethylpaullone or 3-chlorokenpaullone provided good inhibitors. Therefore, several N^5^-aryl substituted derivatives of 3-chlorokenpaullone were synthesized, giving rise to trypanocidal compounds with improved inhibitory activity against trypanosoma TryS [85,147]. Another set of N^5^-substituted paullones with different substituents, including acetic acid and its ester derivatives, acetamides, *N*-(2-aminoethyl)acetamides, or *N*-(4aminobutyl)acetamide (*N*-acetyl putrescine), were synthesized and tested against *L. infantum* TryS. Several of the new derivatives showed good inhibitory properties behaving as uncompetitive enzyme inhibitors and resulted in improved potency and selectivity against *L. braziliensis* and *L. infantum* [148]. Recently, it has been reported that the indole moiety of N^5^-substituted paullones is essential for keeping the TryS-inhibitory and antitrypanosomal activity of this type of compound [149].

#### 3.2.2. Other Compounds Inhibiting TryS

Studies of some natural compounds, such as conessine, tomatine, uvaol, and betulin, have indicated that despite their mild antileishmanial effect, they behaved as *L. donovani* TryS competitive inhibitors towards spermidine and complex inhibitors towards glutathione and ATP [150]. The sesquiterpene lactone cynaropicrin (Figure 7), found in artichokes (*Cynara scolymus* L.) and some species of cornflowers (*Centaurea* spp.), was reported to possess a trypanocidal effect and to inhibit the proliferation of *T. b. rhodesiense* in the acute mouse model. This molecule binds in a covalent manner to reduced glutathione and trypanothione, thus depleting their intracellular pools in *T. brucei*, and although it was a good inhibitor of ornithine decarboxylase, it behaved as a weak inhibitor of TryS and TryR at very high concentrations [151]. Using an in vitro HTS assay, different compounds were reported to efficiently inhibit TryS, including DDD66604 (prochlorperazine) (Figure 7) and three series of compounds that displayed mixed, uncompetitive, and allosteric-type inhibition regarding spermidine, ATP, and glutathione. Representative compounds of these series were able to inhibit the growth of bloodstream *T. brucei* in vitro at the micromolar range, and the addition of the lead indazole compound DDD86243 (Figure 7) to *T. brucei* cultures decreased intracellular trypanothione levels, thus confirming the on-target action of this molecule to inhibit TryS [152]. With a similar core structure to DDD86243, the indazole compound DDU86439 (Figure 7) (*N*-(3-(dimethylamino)propyl)-2-(3-(3-fluorophenyl)-1*H*-indazol-1-yl)acetamide was identified as a potent inhibitor (nanomolar range) of the recombinant *T. brucei* TryS during an HTS campaign. This inhibitor reduced intracellular T[SH]_2_ levels with good potency against bloodstream trypanosomes [84]. Therefore, optimization of the hit indazole series gave rise to compounds that displayed improved in vitro inhibitory potencies but failed in showing sub-micromolar potency as trypanocidal compounds due to the low levels of trypanothione required for parasite viability [153].

Using a computational screening process for inhibitors against redox enzymes of *Leishmania*, oxabicyclonanones were identified as a new class of inhibitors of TryS and TryR. Compound PS-203 (4-(4,4,8-Trimethyl-7-oxo-3-oxabicyclo [3.3.1]non-2-yl)-benzoic acid methyl ester) (Figure 7) was the best molecule in terms of antileishmanial activity (low micromolar range EC_50_), and it induced increased ROS and apoptotic-like death of the parasite due to a competitive inhibition pattern with both TryS and TryR [154].

Benítez and coworkers identified, in addition to MOL2008 (see above), the diamine derivative EAP1-47 (N1,N10-bis(4-isopropylbenzyl) decane-1,10-diamine) (Figure 7) from a library of 144 compounds screened against TryS from *T. brucei*, *T. cruzi*, and *L. infantum*. This compound interfered with T(SH)_2_ biosynthesis and affected the proliferation of *T. brucei* at sub-micromolar concentrations, although other molecular targets are also affected by this molecule [146].

Buthionine sulfoximine (BSO) (Figure 7), the potent inhibitor of γ-glutamylcysteine synthetase [155], was found to partially inhibit recombinant TryS, likely due to the binding of BSO at the glutathione site, and it showed mainly cytostatic effects, thereby suggesting the synthesis of BSO analogs with improved inhibitory effects [156].

From a large library of 51624 compounds screened against *T. brucei* TryS, several hits were found to target the *L. infantum* and *T. cruzi* enzymes [157]. One of these hits, the selenorganic compound Ebselen (2-Phenyl-1,2-benzoselenazol-3(2H)-one) (Figure 7), displayed multi-species TryS inhibitory activity at low micromolar concentrations and anti-proliferative action against *Trypanosma* by rapidly inducing the generation of an intracellular oxidative milieu. Ebselen gave rise to an irreversible modification on a highly conserved cysteine residue from the synthetase domain of TryS acting as a slow-binding inhibitor. In the same study, the authors also reported that the most potent *T. brucei* TryS inhibitor was a singleton containing an adamantine moiety (compound 17) (Figure 7), which behaved as a non-covalent, non-competitive (with any of the substrates) inhibitor [157]. 

After the screening of a library including 144 molecules, several *L. infantum* TryS inhibitors were selected [158]. Two of them, compound 1 (a triazole-phenyl-thiazole) and compound 3 (amphiphile with polyamino moiety), showed a competitive inhibition pattern of *L. infantum* TryS, with a proposed binding site for both inhibitors overlapping the polyamine site, and with compound 1 also occupying part of the ATP site. Another molecule, a polyphenol–carbohydrate hybrid (compound 4), acted as a mixed hyperbolic inhibitor, whereas compound 5 (benzylammonium polar head) (Figure 7) showed kinetic behavior not compatible with a competitive mechanism, and it was the most potent candidate with leishmanicidal activity in the sub-micromolar range [158].

Compound TS001 (Figure 7), containing a pyrrolthiazole-amide group, was selected from an HTS assay used to screen a library of 35,040 compounds, as in addition to inhibiting the recombinant *L. major* TryS, it provided the highest activity against *L. major* and *L. donovani* promastigotes (but not against amastigotes) and *T. brucei brucei* [159]. 

## 4. Conclusions

Since the discovery of trypanothione and its metabolism in 1985, this compound has been of interest to specialists in chemical medicine due to its specificity and unique metabolism in trypanosomatids, which does not exist in its host. The two unique enzymes of the trypanothione metabolism, TryR and TryS, have been studied as targets in several screening programs aimed at finding compounds that effectively inhibit them. Both enzymes have been validated as targets in the three pathogenic trypanosomatids, where defective mutants in both activities led to an increase in the oxidative stress of the microorganism and death. In addition, participation of spermidine in the composition of trypanothione is also relevant, as biosynthesis of this polyamine had been pointed out as a target by a multitude of works related to the discovery of antitrypanosomatid drugs. However, despite the multitude of HTS studies testing compounds targeting recombinant enzymes of pathogenic trypanosomes, few compounds inhibiting the enzymes at sub-micromolar concentrations have been able to prevent parasite growth in vitro or ex vivo, and few have had any impact in experimental animal studies. Apart from the pentavalent antimony derivatives (glucantime and pentostam, used in the clinical treatment of visceral leishmaniasis) and some antiparasitic drugs with a nitroheterocyclic structure, no compound inhibiting these enzymes has reached the clinical stages of drug discovery. Despite these discouraging results, this unique free radical elimination system in trypanosomatids remains an attractive target and inspiration for the synthesis of new compounds or the repositioning of existing ones, thus contributing to the replacement of the obsolete molecules currently treating diseases caused by trypanosomatids.

## Data Availability

Not applicable.

## References

[1] Engels D., Zhou X.N. (2020). Neglected tropical diseases: An effective global response to local poverty-related disease priorities. Infect. Dis. Poverty.

[2] Lukeš J., Butenko A., Hashimi H., Maslov D.A., Votýpka J., Yurchenko V. (2018). Trypanosomatids Are Much More than Just Trypanosomes: Clues from the Expanded Family Tree. Trends Parasitol..

[3] Lin Y., Fang K., Zheng Y., Wang H.L., Wu J. (2022). Global burden and trends of neglected tropical diseases from 1990 to 2019. J. Travel Med..

[4] WHO. https://www.who.int/health-topics/neglected-tropical-diseases#tab=tab_1.

[5] Feasey N., Wansbrough-Jones M., Mabey D.C., Solomon A.W. (2010). Neglected tropical diseases. Br. Med. Bull..

[6] Tidman R., Abela-Ridder B., de Castañeda R.R. (2021). The impact of climate change on neglected tropical diseases: A systematic review. Trans. R. Soc. Trop. Med. Hyg..

[7] Irigoín F., Cibils L., Comini M.A., Wilkinson S.R., Flohé L., Radi R. (2008). Insights into the redox biology of *Trypanosoma cruzi*: Trypanothione metabolism and oxidant detoxification. Free Radic. Biol. Med..

[8] Santi A.M.M., Murta S.M.F. (2022). Antioxidant defence system as a rational target for Chagas disease and Leishmaniasis chemotherapy. Mem. Inst. Oswaldo Cruz.

[9] de Fuentes-Vicente J.A., Gutiérrez-Cabrera A.E., Flores-Villegas A.L., Lowenberger C., Benelli G., Salazar-Schettino P.M., Córdoba-Aguilar A. (2018). What makes an effective Chagas disease vector? Factors underlying *Trypanosoma cruzi*-triatomine interactions. Acta Trop..

[10] Carter Y.L., Juliano J.J., Montgomery S.P., Qvarnstrom Y. (2012). Acute Chagas disease in a returning traveler. Am. J. Trop. Med. Hyg..

[11] de Sousa A.S., Vermeij D., Ramos A.N., Luquetti A.O. (2024). Chagas disease. Lancet.

[12] Nunes M.C., Dones W., Morillo C.A., Encina J.J., Ribeiro A.L. (2013). Council on Chagas Disease of the Interamerican Society of Cardiology. Chagas disease: An overview of clinical and epidemiological aspects. J. Am. Coll. Cardiol..

[13] Echavarría N.G., Echeverría L.E., Stewart M., Gallego C., Saldarriaga C. (2021). Chagas Disease: Chronic Chagas Cardiomyopathy. Curr. Probl. Cardiol..

[14] Rassi A., de Rezende J.M. (2012). Tripanosomiasis americana (enfermedad de Chagas). Infect. Dis. Clin. N. Am..

[15] Pérez-Molina J.A., Molina I. (2018). Chagas disease. Lancet.

[16] Mills R.M. (2020). Chagas Disease: Epidemiology and Barriers to Treatment. Am. J. Trop. Med. Hyg..

[17] Franco J.R., Simarro P.P., Diarra A., Jannin J.G. (2014). Epidemiology of human African trypanosomiasis. Clin. Epidemiol..

[18] Bottieau E., Clerinx J. (2019). Human African Trypanosomiasis: Progress and Stagnation. Infect. Dis. Clin. N. Am..

[19] Ortiz-Martínez Y., Kouamé M.G., Bongomin F., Lakoh S., Henao-Martínez A.F. (2023). Human African Trypanosomiasis (Sleeping Sickness)-Epidemiology, Clinical Manifestations, Diagnosis, Treatment, and Prevention. Curr. Trop. Med. Rep..

[20] Bemba I., Bamou R., Lenga A., Okoko A., Awono-Ambene P., Antonio-Nkondjio C. (2022). Review of the Situation of Human African Trypanosomiasis in the Republic of Congo From the 1950s to 2020. J. Med. Entomol..

[21] Jamabo M., Mahlalela M., Edkins A.L., Boshoff A. (2023). Tackling sleeping sickness: Current and promising therapeutics and treatment strategies. Int. J. Mol. Sci..

[22] Pays E., Radwanska M., Magez S. (2023). The Pathogenesis of African Trypanosomiasis. Annu. Rev. Pathol..

[23] Lejon V., Bentivoglio M., Franco J.R., García H.H., Tanowitz H.B., Del Brutto O.H. (2013). Human African trypanosomiasis. Handbook of Clinical Neurology.

[24] Büscher P., Cecchi G., Jamonneau V., Priotto G. (2017). Human African trypanosomiasis. Lancet.

[25] Kennedy P.G.E. (2019). Update on human African trypanosomiasis (sleeping sickness). J. Neurol..

[26] Laohasinnarong D., Misra G., Srivastava V. (2020). Sleeping sickness. Molecular Advancements in Tropical Diseases Drug Discovery.

[27] Becvar T., Vojtkova B., Siriyasatien P., Votipka J., Modry D., Jahn P., Bates P., Carpintero S., Volf P., Sadlova J. (2021). Experimental transmission of *Leishmania* (*Mundinia*) parasites by biting midges (Diptera: Ceratopogonidae). PLoS Pathog..

[28] Cecílio P., Cordeiro-da-Silva A., Oliveira F. (2022). Sand flies: Basic information on the vectors of leishmaniasis and their interactions with *Leishmania* parasites. Commun. Biol..

[29] Munstermann L.E., Mullen G.R., Durden L.A. (2019). Phlebotomine and sand flies and moth flies (Psychodidae). Medical and Veterinary Entomology.

[30] Sunyoto T., Potet J., Boelaert M. (2017). Visceral leishmaniasis in Somalia: A review of epidemiology and access to care. PLoS Negl. Trop. Dis..

[31] Sasidharan S., Saudagar P. (2021). Leishmaniasis: Where are we and where are we heading?. Parasitol. Res..

[32] de Vries H.J.C., Schallig H.D. (2022). Cutaneous Leishmaniasis: A 2022 Updated Narrative Review into Diagnosis and Management Developments. Am. J. Clin. Dermatol..

[33] Sachdeva H., Sharma M. (2016). Clinical manifestations of leishmaniasis: A Review. Int. J. Adv. Res. Sci. Eng. Technol..

[34] Mann S., Frasca K., Scherrer S., Henao-Martínez A.F., Newman S., Ramanan P., Suarez J.A. (2021). A Review of Leishmaniasis: Current Knowledge and Future Directions. Curr. Trop. Med. Rep..

[35] de Almeida J.V., de Souza C.F., Fuzari A.A., Joya C.A., Valdivia H.O., Bartholomeu D.C., Brasil R.P. (2021). Diagnosis and identification of *Leishmania* species in patients with cutaneous leishmaniasis in the state of Roraima, Brazil’s Amazon Region. Parasites Vectors.

[36] Abadías-Granado I., Diago A., Cerro P.A., Palma-Ruiz A.M., Gilaberte Y. (2021). Leishmaniasis cutánea y mucocutánea. Actas Dermo-Sifiliogr..

[37] Akilov O.E., Khachemoune A., Hasan T. (2007). Clinical manifestations and classification of Old World cutaneous leishmaniasis. Int. J. Dermatol..

[38] Gurel M.S., Tekin B., Uzun S. (2020). Cutaneous leishmaniasis: A great imitator. Clin. Dermatol..

[39] Alcover M.M., Rocamora V., Ribas A., Fisa R., Riera C. (2023). Underestimation of Human Cutaneous Leishmaniasis Caused by *Leishmania infantum* in an Endemic Area of the Mediterranean Basin (Balearic Islands). Microorganisms.

[40] Eichenberger A., Buechi A.E., Neumayr A., Hatz C., Rauch A., Huguenot M., Diamantis-Karamitopoulou E., Staehelin C. (2017). A severe case of visceral leishmaniasis and liposomal amphotericin B treatment failure in an immunosuppressed patient 15 years after exposure. BMC Infect. Dis..

[41] Balaña-Fouce R., Pérez-Pertejo M.Y., Domínguez-Asenjo B., Gutiérrez-Corbo C., Reguera R.M. (2019). Walking a tightrope: Drug discovery in visceral leishmaniasis. Drug Discov. Today.

[42] Dominguez-Asenjo B., Gutierrez-Corbo C., Perez-Pertejo Y., Iborra S., Balana-Fouce R., Reguera R.M. (2021). Bioluminescent Imaging Identifies Thymus, As Overlooked Colonized Organ, in a Chronic Model of *Leishmania donovani* Mouse Visceral Leishmaniasis. ACS Infect. Dis..

[43] Lekic N., Tadic B., Djordjevic V., Basaric D., Micev M., Vucelic D., Mitrovic M., Grubor N. (2022). Splenectomy for Visceral Leishmaniasis Out of an Endemic Region: A Case Report and Literature Review. Medicina.

[44] Costa C.H.N., Chang K.P., Costa D.L., Cunha F.V.M. (2023). From Infection to Death: An Overview of the Pathogenesis of Visceral Leishmaniasis. Pathogens.

[45] Volpedo G., Pachezo-Fernandez T., Holcomb E.A., Cipriano N., Cox B., Satoskar A.R. (2021). Mechanisms of Immunopathogenesis in Cutaneous Leishmaniasis And Post Kala-azar Dermal Leishmaniasis (PKDL). Front. Cell. Infect. Microbiol..

[46] Lutje V., Probyn K., Seixas J., Bergman H., Villanueva G. (2021). Chemotherapy for second-stage human African trypanosomiasis: Drugs in use. Cochrane Database Syst. Rev..

[47] García-Estrada C., Pérez-Pertejo Y., Domínguez-Asenjo B., Holanda V.N., Murugesan S., Martínez-Valladares M., Balaña-Fouce R., Reguera R.M. (2023). Further investigations of nitroheterocyclic compounds as potential antikinetoplastid drug candidates. Biomolecules.

[48] Reguera R.M., Pérez-Pertejo Y., Gutiérrez-Corbo C., Martínez-Valladares M., Balaña-Fouce R. (2019). Current and promising novel drug candidates against visceral leishmaniasis. Pure Appl. Chem..

[49] Lago A.S.D., Nascimento M., Carvalho A.M., Lago N., Silva J., Queiroz J.R., Carvalho L.P., Schriefer A., Wilson M., Machado P. (2018). The elderly responds to antimony therapy for cutaneous leishmaniasis similarly to young patients but have severe adverse reactions. Am. J. Trop. Med. Hyg..

[50] Chakravarty J., Sundar S. (2019). Current and emerging medications for the treatment of leishmaniasis. Expert Opin. Pharmacother..

[51] Palić S., Beijnen J.H., Dorlo T.P.C. (2022). An update on the clinical pharmacology of miltefosine in the treatment of leishmaniasis. Int. J. Antimicrob. Agents.

[52] Reguera R.M., Calvo-Álvarez E., Álvarez-Velilla R., Balaña-Fouce R. (2014). Target-based vs. phenotypic screenings in *Leishmania* drug discovery: A marriage of convenience or a dialogue of the deaf?. Int. J. Parasitol. Drugs Drug Resist..

[53] Raj S., Sasidharan S., Balaji S.N., Saudagar P. (2020). An overview of biochemically characterized drug targets in metabolic pathways of *Leishmania* parasite. Parasitol. Res..

[54] Kourbeli V., Chontzopoulou E., Moschovou K., Pavlos D., Mavromoustakos T., Papanastasiou I.P. (2021). An overview on target-based drug design against kinetoplastid protozoan infections: Human African trypanosomiasis, Chagas disease and leishmaniases. Molecules.

[55] Jain S., Sahu U., Kumar A., Khare P. (2022). Metabolic pathways of *Leishmania* parasite: Source of pertinent drug targets and potent drug candidates. Pharmaceutics.

[56] Pérez-Pertejo Y., García-Estrada C., Martínez-Valladares M., Murugesan S., Reguera R.M., Balaña-Fouce R. (2024). Polyamine Metabolism for Drug Intervention in Trypanosomatids. Pathogens.

[57] Ali V., Behera S., Nawaz A., Equbal A., Pandey K. (2022). Unique thiol metabolism in trypanosomatids: Redox homeostasis and drug resistance. Adv. Parasitol..

[58] Passos A.O., Assis L.H.C., Ferri Y.G., da Silva V.L., da Silva M.S., Cano M.I.N., Wang Z. (2022). The Trypanosomatids Cell Cycle: A Brief Report. Cell-Cycle Synchronization.

[59] Bates P.A. (2007). Transmission of *Leishmania* metacyclic promastigotes by phlebotomine sand flies. Int. J. Parasitol..

[60] Sorci G., Faivre B. (2009). Inflammation and oxidative stress in vertebrate host-parasite systems. Philos. Trans. R. Soc. B Biol. Sci..

[61] Mesías A.C., Garg N.J., Zago M.P. (2019). Redox balance keepers and possible cell functions managed by redox homeostasis in *Trypanosoma cruzi*. Front. Cell. Infect. Microbiol..

[62] Turrens J.F. (2003). Mitochondrial formation of reactive oxygen species. J. Physiol..

[63] Zabala-Peñafiel A., Cysne-Finkelstein L., Conceição-Silva F., Fagundes A., Miranda L.F.C., Souza-Silva F., Brandt A.A.M.L., Dias-Lopes G., Alves C.R. (2022). Novel Insights Into *Leishmania* (*Viannia*) *braziliensis* In Vitro Fitness Guided by Temperature Changes Along with Its Subtilisins and Oligopeptidase. Front. Cell. Infect. Microbiol..

[64] da Silva M.S., Segatto M., Pavani R.S., Gutierrez-Rodrigues F., Bispo V.D., de Medeiros M.H., Calado R., Elias M.C., Cano M.I.N. (2017). Consequences of acute oxidative stress in *Leishmania amazonensis*: From telomere shortening to the selection of the fittest parasites. Biochim. Biophys. Acta (BBA)-Mol. Cell Res..

[65] Krauth-Siegel R.L., Comini M.A. (2008). Redox control in trypanosomatids, parasitic protozoa with trypanothione-based thiol metabolism. Biochim. Biophys. Acta (BBA)-Gen. Subj..

[66] Turrens J.F. (2004). Oxidative stress and antioxidant defenses: A target for the treatment of diseases caused by parasitic protozoa. Mol. Asp. Med..

[67] Flohé L., Hecht H.J., Steinert P. (1999). Glutathione and trypanothione in parasitic hydroperoxide metabolism. Free Radic. Biol. Med..

[68] Castro H., Romao S., Carvalho S., Teixeira F., Sousa C., Tomas A.M. (2010). Mitochondrial Redox Metabolism in Trypanosomatids Is Independent of Tryparedoxin Activity. PLoS ONE.

[69] Fairlamb A.H., Blackburn P., Ulrich P., Chait B.T., Cerami A. (1985). Trypanothione: A novel bis(glutathionyl)spermidine cofactor for glutathione reductase in trypanosomatids. Science.

[70] Krauth-Siegel R.L., Meiering S.K., Schmidt H. (2003). The parasite-specific trypanothione metabolism of *Trypanosoma* and *Leishmania*. Biol. Chem..

[71] Manta B., Comini M., Medeiros A., Hugo M., Radi R. (2013). Trypanothione: A unique bis-glutathionyl derivative in trypanosomatids. Biochim. Biophys. Acta (BBA)-Gen. Subj..

[72] Hillebrand H., Schmidt A., Krauth-Siegel R.L. (2003). A second class of peroxidases linked to the trypanothione metabolism. J. Biol. Chem..

[73] Krieger S., Schwarz W., Ariyanayagam M.R., Fairlamb A.H., Krauth-Siegel R.L., Clayton C. (2000). Trypanosomes lacking trypanothione reductase are avirulent and show increased sensitivity to oxidative stress. Mol. Microbiol..

[74] Singh B.K., Sarkar N., Jagannadham M.V., Dubey V.K. (2008). Modeled structure of trypanothione reductase of *Leishmania infantum*. BMB Rep..

[75] Castro H., Tomás A.M. (2008). Peroxidases of trypanosomatids. Antioxid. Redox Signal..

[76] Colotti G., Baiocco P., Fiorillo A., Boffi A., Poser E., Chiaro F.D., Ilari A. (2013). Structural insights into the enzymes of the trypanothione pathway: Targets for antileishmaniasis drugs. Future Med. Chem..

[77] Oza S.L., Tetaud E., Ariyanayagam M.R., Warnon S.S., Fairlamb A.H. (2002). A single enzyme catalyses formation of Trypanothione from glutathione and spermidine in *Trypanosoma cruzi*. J. Biol. Chem..

[78] Phillips M.A. (2018). Polyamines in protozoan pathogens. J. Biol. Chem..

[79] Reguera R.M., Tekwani B.L., Balaña-Fouce R. (2005). Polyamine transport in parasites: A potential target for new antiparasitic drug development. Comp. Biochem. Physiol. Part C Toxicol. Pharmacol..

[80] Balaña-Fouce R., Calvo-Álvarez E., Álvarez-Velilla R., Prada C.F., Pérez-Pertejo Y., Reguera R.M. (2012). Role of trypanosomatid’s arginase in polyamine biosynthesis and pathogenesis. Mol. Biochem. Parasitol..

[81] Reguera R.M., Redondo C.M., Pérez-Pertejo Y., Balaña-Fouce R. (2007). S-Adenosylmethionine in protozoan parasites: Functions, synthesis and regulation. Mol. Biochem. Parasitol..

[82] Flohé L. (2012). The trypanothione system and the opportunities it offers to create drugs for the neglected kinetoplast diseases. Biotechnol. Adv..

[83] Sousa A.F., Gomes-Alves A.G., Benítez D., Comini M.A., Flohé L., Jaeger T., Passos J., Stuhlmann F., Tomás A.M., Castro H. (2014). Genetic and chemical analyses reveal that trypanothione synthetase but not glutathionylspermidine synthetase is essential for *Leishmania infantum*. Free Radic. Biol. Med..

[84] Wyllie S., Oza S.L., Patterson S., Spinks D., Thompson S., Fairlamb A.H. (2009). Dissecting the essentiality of the bifunctional trypanothione synthetase-amidase in *Trypanosoma brucei* using chemical and genetic methods. Mol. Microbiol..

[85] Mesías A.C., Sasoni N., Arias D.G., Pérez Brandán C., Orban O.C.F., Kunick C., Robello C., Comini M.A., Garg N.J., Zago M.P. (2019). Trypanothione synthetase confers growth, survival advantage and resistance to anti-protozoal drugs in *Trypanosoma cruzi*. Free Radic. Biol. Med..

[86] Fyfe P.K., Oza S.L., Fairlamb A.H., Hunter W.N. (2008). *Leishmania* trypanothione synthetase-amidase structure reveals a basis for regulation of conflicting synthetic and hydrolytic activities. J. Biol. Chem..

[87] Oza S.L., Ariyanayagam M.R., Fairlamb A.H. (2002). Characterization of recombinant glutathionylspermidine synthetase/amidase from *Crithidia fasciculata*. Biochem. J..

[88] Bond C.S., Zhang Y., Berriman M., Cunningham M.L., Fairlamb A.H., Hunter W.N. (1999). Crystal structure of *Trypanosoma cruzi* trypanothione reductase in complex with trypanothione, and the structure-based discovery of new natural product inhibitors. Structure.

[89] Baiocco P., Poce G., Alfonso S., Cocozza M., Porretta G.C., Colotti G., Biava M., Moraca F., Botta M., Yardley V. (2013). Inhibition of *Leishmania infantum* trypanothione reductase by azole-based compounds: A comparative analysis with its physiological substrate by X-ray crystallography. ChemMedChem.

[90] Jones D.C., Ariza A., Chow W.H., Oza S.L., Fairlamb A.H. (2010). Comparative structural, kinetic and inhibitor studies of *Trypanosoma brucei* trypanothione reductase with *T. cruzi*. Mol. Biochem. Parasitol..

[91] Battista T., Colotti G., Ilari A., Fiorillo A. (2020). Targeting Trypanothione Reductase, a Key Enzyme in the Redox Trypanosomatid Metabolism, to Develop New Drugs against Leishmaniasis and Trypanosomiases. Molecules.

[92] Piñeyro M.D., Arias D., Parodi-Talice A., Guerrero S., Robello C. (2021). Trypanothione Metabolism as Drug Target for Trypanosomatids. Curr. Pharm. Des..

[93] Kumar A., Nimsarkar P., Singh S. (2022). Probing the Interactions Responsible for the Structural Stability of Trypanothione Reductase Through Computer Simulation and Biophysical Characterization. Protein J..

[94] Madia V.N., Ialongo D., Patacchini E., Exertier C., Antonelli L., Colotti G., Messore A., Tudino V., Saccoliti F., Scipione L. (2023). Inhibition of *Leishmania infantum* Trypanothione Reductase by New Aminopropanone Derivatives Interacting with the NADPH Binding Site. Molecules.

[95] Saccoliti F., Di Santo R., Costi R. (2020). Recent Advancement in the Search of Innovative Antiprotozoal Agents Targeting Trypanothione Metabolism. ChemMedChem.

[96] Jacoby E.M., Schlichting I., Lantwin C.B., Kabsch W., Krauth-Siegel R.L. (1996). Crystal structure of the *Trypanosoma cruzi* trypanothione reductase mepacrine complex. Proteins.

[97] Bonse S., Santelli-Rouvier C., Barbe J., Krauth-Siegel R.L. (1999). Inhibition of *Trypanosoma cruzi* trypanothione reductase by acridines: Kinetic studies and structure-activity relationships. J. Med. Chem..

[98] Saravanamuthu A., Vickers T.J., Bond C.S., Peterson M.R., Hunter W.N., Fairlamb A.H. (2004). Two interacting binding sites for quinacrine derivatives in the active site of trypanothione reductase: A template for drug design. J. Biol. Chem..

[99] Hossain M.U., Oany A.R., Ahmad S.A.I., Hasan M.A., Khan M.A., Siddikey M.A.A. (2016). Identification of potential inhibitor and enzyme-inhibitor complex on trypanothione reductase to control Chagas disease. Comput. Biol. Chem..

[100] Chibale K., Haupt H., Kendrick H., Yardley V., Saravanamuthu A., Fairlamb A.H., Croft S.L. (2001). Antiprotozoal and cytotoxicity evaluation of sulfonamide and urea analogues of quinacrine. Bioorg. Med. Chem. Lett..

[101] Eberle C., Burkhard J.A., Stump B., Kaiser M., Brun R., Krauth-Siegel R.L., Diederich F. (2009). Synthesis, inhibition potency, binding mode, and antiprotozoal activities of fluorescent inhibitors of trypanothione reductase based on mepacrine-conjugated diaryl sulfide scaffolds. ChemMedChem.

[102] Richardson J.L., Nett I.R., Jones D.C., Abdille M.H., Gilbert I.H., Fairlamb A.H. (2009). Improved tricyclic inhibitors of trypanothione reductase by screening and chemical synthesis. ChemMedChem.

[103] Patterson S., Jones D.C., Shanks E.J., Frearson J.A., Gilbert I.H., Wyatt P.G., Fairlamb A.H. (2009). Synthesis and evaluation of 1-(1-(Benzo[b]thiophen-2-yl)cyclohexyl)piperidine (BTCP) analogues as inhibitors of trypanothione reductase. ChemMedChem.

[104] Eberle C., Lauber B.S., Fankhauser D., Kaiser M., Brun R., Krauth-Siegel R.L., Diederich F. (2011). Improved inhibitors of trypanothione reductase by combination of motifs: Synthesis, inhibitory potency, binding mode, and antiprotozoal activities. ChemMedChem.

[105] De Gasparo R., Brodbeck-Persch E., Bryson S., Hentzen N.B., Kaiser M., Pai E.F., Krauth-Siegel R.L., Diederich F. (2018). Biological Evaluation and X-ray Co-crystal Structures of Cyclohexylpyrrolidine Ligands for Trypanothione Reductase, an Enzyme from the Redox Metabolism of *Trypanosoma*. ChemMedChem.

[106] De Gasparo R., Halgas O., Harangozo D., Kaiser M., Pai E.F., Krauth-Siegel R.L., Diederich F. (2019). Targeting a Large Active Site: Structure-Based Design of Nanomolar Inhibitors of *Trypanosoma brucei* Trypanothione Reductase. Chemistry.

[107] Holloway G.A., Charman W.N., Fairlamb A.H., Brun R., Kaiser M., Kostewicz E., Novello P.M., Parisot J.P., Richardson J., Street I.P. (2009). Trypanothione reductase high-throughput screening campaign identifies novel classes of inhibitors with antiparasitic activity. Antimicrob. Agents Chemother..

[108] Patterson S., Alphey M.S., Jones D.C., Shanks E.J., Street I.P., Frearson J.A., Wyatt P.G., Gilbert I.H., Fairlamb A.H. (2011). Dihydroquinazolines as a novel class of *Trypanosoma brucei* trypanothione reductase inhibitors: Discovery, synthesis, and characterization of their binding mode by protein crystallography. J. Med. Chem..

[109] Matadamas-Martínez F., Hernández-Campos A., Téllez-Valencia A., Vázquez-Raygoza A., Comparán-Alarcón S., Yépez-Mulia L., Castillo R. (2019). *Leishmania mexicana* Trypanothione Reductase Inhibitors: Computational and Biological Studies. Molecules.

[110] Turcano L., Battista T., De Haro E.T., Missineo A., Alli C., Paonessa G., Colotti G., Harper S., Fiorillo A., Ilari A. (2020). Spiro-containing derivatives show antiparasitic activity against *Trypanosoma brucei* through inhibition of the trypanothione reductase enzyme. PLoS Negl. Trop. Dis..

[111] Saccoliti F., Angiulli G., Pupo G., Pescatori L., Madia V.N., Messore A., Colotti G., Fiorillo A., Scipione L., Gramiccia M. (2017). Inhibition of *Leishmania infantum* trypanothione reductase by diaryl sulfide derivatives. J. Enzym. Inhib. Med. Chem..

[112] Colotti G., Saccoliti F., Gramiccia M., Di Muccio T., Prakash J., Yadav S., Dubey V.K., Vistoli G., Battista T., Mocci S. (2020). Structure-guided approach to identify a novel class of anti-leishmaniasis diaryl sulfide compounds targeting the trypanothione metabolism. Amino Acids.

[113] Etxebeste-Mitxeltorena M., Plano D., Espuelas S., Moreno E., Aydillo C., Jiménez-Ruiz A., Soriano J.C.G., Sanmartín C. (2020). New Amides Containing Selenium as Potent Leishmanicidal Agents Targeting Trypanothione Reductase. Antimicrob. Agents Chemother..

[114] Aguilera-Venegas B., Olea-Azar C., Norambuena E., Arán V.J., Mendizábal F., Lapier M., Maya J.D., Kemmerling U., López-Muñoz R. (2011). ESR, electrochemical, molecular modeling and biological evaluation of 4-substituted and 1,4-disubstituted 7-nitroquinoxalin-2-ones as potential anti-*Trypanosoma cruzi* agents. Spectrochim. Acta Part A Mol. Biomol. Spectrosc..

[115] Battista T., Federico S., Brogi S., Pozzetti L., Khan T., Butini S., Ramunno A., Fiorentino E., Orsini S., Di Muccio T. (2022). Optimization of Potent and Specific Trypanothione Reductase Inhibitors: A Structure-Based Drug Discovery Approach. ACS Infect. Dis..

[116] Exertier C., Salerno A., Antonelli L., Fiorillo A., Ocello R., Seghetti F., Caciolla J., Uliassi E., Masetti M., Fiorentino E. (2024). Fragment Merging, Growing, and Linking Identify New Trypanothione Reductase Inhibitors for Leishmaniasis. J. Med. Chem..

[117] Cunningham M.L., Fairlamb A.H. (1995). Trypanothione reductase from *Leishmania donovani*. Purification, characterisation and inhibition by trivalent antimonials. Eur. J. Biochem..

[118] Baiocco P., Colotti G., Franceschini S., Ilari A. (2009). Molecular basis of antimony treatment in leishmaniasis. J. Med. Chem..

[119] Baiocco P., Ilari A., Ceci P., Orsini S., Gramiccia M., Di Muccio T., Colotti G. (2010). Inhibitory Effect of Silver Nanoparticles on Trypanothione Reductase Activity and *Leishmania infantum* Proliferation. ACS Med. Chem. Lett..

[120] Colotti G., Ilari A., Fiorillo A., Baiocco P., Cinellu M.A., Maiore L., Scaletti F., Gabbiani C., Messori L. (2013). Metal-based compounds as prospective antileishmanial agents: Inhibition of trypanothione reductase by selected gold complexes. ChemMedChem.

[121] Ilari A., Baiocco P., Messori L., Fiorillo A., Boffi A., Gramiccia M., Di Muccio T., Colotti G. (2012). A gold-containing drug against parasitic polyamine metabolism: The X-ray structure of trypanothione reductase from *Leishmania infantum* in complex with auranofin reveals a dual mechanism of enzyme inhibition. Amino Acids.

[122] Bonse S., Richards J.M., Ross S.A., Lowe G., Krauth-Siegel R.L. (2000). (2,2’:6’,2’’-Terpyridine)platinum(II) complexes are irreversible inhibitors of *Trypanosoma cruzi* trypanothione reductase but not of human glutathione reductase. J. Med. Chem..

[123] Otero L., Vieites M., Boiani L., Denicola A., Rigol C., Opazo L., Olea-Azar C., Maya J.D., Morello A., Krauth-Siegel R.L. (2006). Novel antitrypanosomal agents based on palladium nitrofurylthiosemicarbazone complexes: DNA and redox metabolism as potential therapeutic targets. J. Med. Chem..

[124] Turcano L., Torrente E., Missineo A., Andreini M., Gramiccia M., Di Muccio T., Genovese I., Fiorillo A., Harper S., Bresciani A. (2018). Identification and binding mode of a novel *Leishmania* Trypanothione reductase inhibitor from high throughput screening. PLoS Negl. Trop. Dis..

[125] Toro M.A., Sánchez-Murcia P.A., Moreno D., Ruiz-Santaquiteria M., Alzate J.F., Negri A., Camarasa M.J., Gago F., Velázquez S., Jiménez-Ruiz A. (2013). Probing the dimerization interface of *Leishmania infantum* trypanothione reductase with site-directed mutagenesis and short peptides. Chembiochem.

[126] de Lucio H., Toro M.A., Camarasa M.J., Velázquez S., Gago F., Jiménez-Ruiz A. (2020). Pseudoirreversible slow-binding inhibition of trypanothione reductase by a protein-protein interaction disruptor. Br. J. Pharmacol..

[127] Revuelto A., Ruiz-Santaquiteria M., de Lucio H., Gamo A., Carriles A.A., Gutiérrez K.J., Sánchez-Murcia P.A., Hermoso J.A., Gago F., Camarasa M.J. (2019). Pyrrolopyrimidine vs. Imidazole-Phenyl-Thiazole Scaff olds in Nonpeptidic Dimerization Inhibitors of *Leishmania infantum* Trypanothione Reductase. ACS Infect. Dis..

[128] Revuelto A., de Lucio H., García-Soriano J.C., Sánchez-Murcia P.A., Gago F., Jiménez-Ruiz A., Camarasa M.J., Velázquez S. (2021). Efficient Dimerization Disruption of *Leishmania infantum* Trypanothione Reductase by Triazole-phenyl-thiazoles. J. Med. Chem..

[129] Uliassi E., Fiorani G., Krauth-Siegel R.L., Bergamini C., Fato R., Bianchini G., Carlos Menéndez J., Molina M.T., López-Montero E., Falchi F. (2017). Crassiflorone derivatives that inhibit *Trypanosoma brucei* glyceraldehyde-3-phosphate dehydrogenase (TbGAPDH) and *Trypanosoma cruzi* trypanothione reductase (TcTR) and display trypanocidal activity. Eur. J. Med. Chem..

[130] González-González A., Sánchez-Sánchez O., Krauth-Siegel R.L., Bolognesi M.L., Gớmez-Escobedo R., Nogueda-Torres B., Vázquez-Jiménez L.K., Saavedra E., Encalada R., Espinoza-Hicks J.C. (2022). In Vitro and In Silico Analysis of New n-Butyl and Isobutyl Quinoxaline-7-carboxylate 1,4-di-N-oxide Derivatives against *Trypanosoma cruzi* as Trypanothione Reductase Inhibitors. Int. J. Mol. Sci..

[131] Espinosa-Bustos C., Ortiz-Pérez M., González-González A., Zarate A.M., Rivera G., Belmont-Díaz J.A., Saavedra E., Cuellar M.A., Vázquez K., Salas C.O. (2022). New Amino Naphthoquinone Derivatives as Anti-*Trypanosoma cruzi* Agents Targeting Trypanothione Reductase. Pharmaceutics.

[132] de Lucio H., García-Marín J., Sánchez-Alonso P., García-Soriano J.C., Toro M.Á., Vaquero J.J., Gago F., Alajarín R., Jiménez-Ruiz A. (2022). Pyridazino-pyrrolo-quinoxalinium salts as highly potent and selective leishmanicidal agents targeting trypanothione reductase. Eur. J. Med. Chem..

[133] González-González A., Vázquez C., Encalada R., Saavedra E., Vázquez-Jiménez L.K., Ortiz-Pérez E., Bolognesi M.L., Rivera G. (2023). Phenothiazine-based virtual screening, molecular docking, and molecular dynamics of new trypanothione reductase inhibitors of *Trypanosoma cruzi*. Mol. Inform..

[134] Henderson G.B., Ulrich P., Fairlamb A.H., Rosenberg I., Pereira M., Sela M., Cerami A. (1988). “Subversive” substrates for the enzyme trypanothione disulfide reductase: Alternative approach to chemotherapy of Chagas disease. Proc. Natl. Acad. Sci. USA.

[135] Morin C., Besset T., Moutet J.C., Fayolle M., Brückner M., Limosin D., Becker K., Davioud-Charvet E. (2008). The aza-analogues of 1,4-naphthoquinones are potent substrates and inhibitors of plasmodial thioredoxin and glutathione reductases and of human erythrocyte glutathione reductase. Org. Biomol. Chem..

[136] Salmon-Chemin L., Buisine E., Yardley V., Kohler S., Debreu M.A., Landry V., Sergheraert C., Croft S.L., Krauth-Siegel R.L., Davioud-Charvet E. (2001). 2- and 3-substituted 1,4-naphthoquinone derivatives as subversive substrates of trypanothione reductase and lipoamide dehydrogenase from *Trypanosoma cruzi*: Synthesis and correlation between redox cycling activities and in vitro cytotoxicity. J. Med. Chem..

[137] Cenas N., Bironaite D., Dickancaite E., Anusevicius Z., Sarlauskas J., Blanchard J.S. (1994). Chinifur, a selective inhibitor and “subversive substrate” for *Trypanosoma congolense* trypanothione reductase. Biochem. Biophys. Res. Commun..

[138] Blumenstiel K., Schöneck R., Yardley V., Croft S.L., Krauth-Siegel R.L. (1999). Nitrofuran drugs as common subversive substrates of *Trypanosoma cruzi* lipoamide dehydrogenase and trypanothione reductase. Biochem. Pharmacol..

[139] Arias D.G., Herrera F.E., Garay A.S., Rodrigues D., Forastieri P.S., Luna L.E., Bürgi M.D., Prieto C., Iglesias A.A., Cravero R.M. (2017). Rational design of nitrofuran derivatives: Synthesis and valuation as inhibitors of *Trypanosoma cruzi* trypanothione reductase. Eur. J. Med. Chem..

[140] Domínguez-Asenjo B., Gutiérrez-Corbo C., Álvarez-Bardón M., Pérez-Pertejo Y., Balaña-Fouce R., Reguera R.M. (2021). Ex Vivo Phenotypic Screening of Two Small Repurposing Drug Collections Identifies Nifuratel as a Potential New Treatment against Visceral and Cutaneous Leishmaniasis. ACS Infect. Dis..

[141] Melcón-Fernandez E., Galli G., García-Estrada C., Balaña-Fouce R., Reguera R.M., Pérez-Pertejo Y. (2023). Miltefosine and Nifuratel Combination: A Promising Therapy for the Treatment of *Leishmania donovani* Visceral Leishmaniasis. Int. J. Mol. Sci..

[142] Alice J.I., Bellera C.L., Benítez D., Comini M.A., Duchowicz P.R., Talevi A. (2021). Ensemble learning application to discover new trypanothione synthetase inhibitors. Mol. Divers..

[143] Leroux A.E., Krauth-Siegel R.L. (2016). Thiol redox biology of trypanosomatids and potential targets for chemotherapy. Mol. Biochem. Parasitol..

[144] Koch O., Jäger T., Flohé L., Selzer P.M., Jäger T., Koch O., Flohé L. (2013). Inhibition of trypanothione synthetase as a therapeutic concept. Trypanosomatid Diseases: Molecular Routes to Drug Discovery.

[145] Maiwald F., Benítez D., Charquero D., Dar M.A., Erdmann H., Preu L., Koch O., Hölscher C., Loaëc N., Meijer L. (2014). 9- and 11-Substituted 4-azapaullones are potent and selective inhibitors of African trypanosoma. Eur. J. Med. Chem..

[146] Benítez D., Medeiros A., Fiestas L., Panozzo-Zenere E.A., Maiwald F., Prousis K.C., Roussaki M., Calogeropoulou T., Detsi A., Jaeger T. (2016). Identification of Novel Chemical Scaffolds Inhibiting Trypanothione Synthetase from Pathogenic Trypanosomatids. PLoS Negl. Trop. Dis..

[147] Orban O.C.F., Korn R.S., Benıtez D., Medeiros A., Preu L., Loaëc N., Meijer L., Koch O., Comini M.A., Kunick C. (2016). 5-Substituted 3-chlorokenpaullone derivatives are potent inhibitors of *Trypanosoma brucei* bloodstream forms. Bioorganic Med. Chem..

[148] Medeiros A., Benítez D., Korn R.S., Ferreira V.C., Barrera E., Carrión F., Pritsch O., Pantano S., Kunick C., de Oliveira C.I. (2020). Mechanistic and biological characterisation of novel N5-substituted paullones targeting the biosynthesis of trypanothione in Leishmania. J. Enzym. Inhib. Med. Chem..

[149] Ihnatenko I., Müller M.J., Orban O.C.F., Lindhof J.C., Benítez D., Ortíz C., Dibello E., Seidl L.L., Comini M.A., Kunick C. (2023). The indole motif is essential for the antitrypanosomal activity of N5-substituted paullones. PLoS ONE.

[150] Saudagar P., Dubey V.K. (2011). Cloning, expression, characterization and inhibition studies on trypanothione synthetase, a drug target enzyme, from *Leishmania donovani*. Biol. Chem..

[151] Zimmermann S., Oufir M., Leroux A., Krauth-Siegel R.L., Becker K., Kaiser M., Brun R., Hamburger M., Adams M. (2013). Cynaropicrin targets the trypanothione redox system in *Trypanosoma brucei*. Bioorg. Med. Chem..

[152] Torrie L.S., Wyllie S., Spinks D., Oza S.L., Thompson S., Harrison J.R., Gilbert I.H., Wyatt P.G., Fairlamb A.H., Frearson J.A. (2009). Chemical validation of trypanothione synthetase: A potential drug target for human trypanosomiasis. J. Biol. Chem..

[153] Spinks D., Torrie L.S., Thompson S., Harrison J.R., Frearson J.A., Read K.D., Fairlamb A.H., Wyatt P.G., Gilbert I.H. (2012). Design, synthesis and biological evaluation of *Trypanosoma brucei* trypanothione synthetase inhibitors. ChemMedChem.

[154] Saudagar P., Saha P., Saikia A.K., Dubey V.K. (2013). Molecular mechanism underlying antileishmanial effect of oxabicyclo [3.3.1]nonanones: Inhibition of key redox enzymes of the pathogen. Eur. J. Pharm. Biopharm..

[155] Griffith O.W. (1982). Mechanism of action, metabolism, and toxicity of buthionine sulfoximine and its higher homologs, potent inhibitors of glutathione synthesis. J. Biol. Chem..

[156] Vázquez C., Mejia-Tlachi M., González-Chávez Z., Silva A., Rodríguez-Zavala J.S., Moreno-Sánchez R., Saavedra E. (2017). Buthionine sulfoximine is a multitarget inhibitor of trypanothione synthesis in *Trypanosoma cruzi*. FEBS Lett..

[157] Benítez D., Franco J., Sardi F., Leyva A., Durán R., Choi G., Yang G., Kim T., Kim N., Heo J. (2022). Drug-like molecules with anti-trypanothione synthetase activity identified by high throughput screening. J. Enzym. Inhib. Med. Chem..

[158] Alcón-Calderón M., de Lucio H., García-Soriano J.C., Revuelto A., de Castro S., López-Gutiérrez C., San-Félix A., Quesada E., Gago F., Camarasa M.J. (2022). Identification of *L. infantum* trypanothione synthetase inhibitors with leishmanicidal activity from a (non-biased) in-house chemical library. Eur. J. Med. Chem..

[159] Phan T.N., Park K.P., Benítez D., Comini M.A., Shum D., No J.H. (2022). Discovery of novel *Leishmania major* trypanothione synthetase inhibitors by high-throughput screening. Biochem. Biophys. Res. Commun..

